# Paraben Levels in an Urban Community of Western Canada

**DOI:** 10.1155/2013/507897

**Published:** 2013-12-17

**Authors:** Stephen J. Genuis, Detlef Birkholz, Luke Curtis, Court Sandau

**Affiliations:** ^1^Faculty of Medicine, University of Alberta, Canada T6K 4C1; ^2^ALS Laboratory Group, Canada T6E 5C1; ^3^Forest Hills, NY 11375, USA

## Abstract

With effective antibacterial and antifungal properties, commercially used parabens are synthetic compounds widely utilized as preservatives in cosmetics, personal care products, pharmaceuticals, and as an additive in some foodstuffs. While long regarded as relatively safe and nontoxic, recent research has demonstrated xenoestrogenic properties of anthropogenic parabens with early evidence that paraben exposure may be linked to breast cancer, thyroid dysfunction, allergy, and obesity. In an attempt to determine the prevalence of paraben exposure in a Canadian urban community, a sample of convenience was done by measuring urinary levels of methyl, ethyl, propyl, butyl, and isobutyl parabens (MP, EP, PP, BP, and IP) in 39 consecutive patients in an Alberta primary care clinic. In 28 female patients including 9 pregnant women, the median urinary levels (in **μ**g/L) were 25.45 for MP, 10.17 for EP, 2.80 for PP, 0.30 for BP, and 0.24 for IP. In 11 male patients, the median urinary levels (in **μ**g/L) were 25.95 for MP, 10.37 for EP, 3.09 for PP, 0.35 for BP, and 0.22 for IP. Especially high urinary paraben levels were reported in a few patients, with the highest urinary concentrations (in **μ**g/L) reported as 966.46 for MP, 220.6 as EP, and 612.73 for PP. It is evident that exposure to assorted parabens is a routine event for many if not most individuals, including pregnant women, in urban Alberta, Canada.

## 1. Introduction

Parabens are a collective term for alkyl esters of parahydroxybenzoic acid (PHBA). With broad antibacterial and antifungal properties, parabens have been used as antimicrobial preservatives in food, beverages, drugs, and personal care products for the past 75 years [1]. Common parabens include methyl paraben (MP), ethyl paraben (EP), propyl paraben (PP), butylparaben (BP), and isobutyl paraben (IP). These compounds are relatively inexpensive and have long been believed to have low levels of toxicity, irritation, and sensitizing potential [1]. With recent evidence that parabens may act as endocrine disrupting chemicals, however, increasing research is underway to delineate potential human health sequelae that may result from exposure to these compounds. In an attempt to determine the prevalence of paraben exposure in one representative urban community in Canada, a sample of convenience was done by measuring urinary levels of common parabens in patients in an Alberta primary care clinic.

### 1.1. Background

With broad spectrum antimicrobial efficacy and chemical stability, parabens have become a popular preservative additive in foodstuffs, cosmetics, and pharmaceuticals. People are potentially exposed to parabens via ingestion, inhalation, and/or dermal absorption [2]. Many products contain parabens including some toothpastes, sunscreens, body lotions, facial lotions and cleansers, mascara, assorted lipsticks, hand soaps, as well as hair products including shampoos, conditioners, sprays, gels, and some food products [3–6].

Human and animal studies have reported that parabens are readily absorbed through the skin [1, 7]. Cosmetic exposure is believed to be the predominant paraben exposure route for most adults in developed nations, with estimates of dermal total paraben internal exposure ranging from 0.03 to 4.13 mg/kg bodyweight/day [8]. Close to half of cosmetics tested in the US contain parabens [3] with MP being the most common agent found and lipstick having the highest paraben concentration [5].

Oral ingestion appears to represent a smaller portion of total paraben exposure, comprising an estimated 0.42% to 5.5% of total paraben exposure in Chinese and US populations [9]. MP and PP are the most commonly used paraben preservatives in food products as well as in pharmaceutical preparations [5, 6]. A study of 267 assorted foodstuffs in Albany, NY reported average total paraben levels of 9.67 ng/mg [2]. The highest mean total levels of parabens were found in beverages and grain products, and the lowest mean total parabens levels were reported in oils/fats, fruits, and fish/shellfish. MP was found at levels above lower detection limit in 91% of samples, while EP was found in 62% samples. Estimated dietary intakes of total parabens in the United States (in ng/kg body weight/day) ranged from 940 in infants less than 1 year old to 307 in adults over age 21 years [2].

Parabens are generally excreted in the urine in a form conjugated with glucuronide or sulfate to make them more water soluble [10]. A study of 100 human adults reported that urinary MP was excreted 5% in free form, 28% as a glucuronide conjugate, and 67% as a sulfate conjugate, while urinary PP was excreted 2% in free form, 43% as a glucuronide conjugate, and 55% as a sulfate conjugate [10]. Some excretion of parabens also occurs through the bile and feces [7].

### 1.2. Parabens and Health Concerns

Previously regarded as safe, parabens have recently drawn concern with increasing evidence of the hormone disrupting potential of these compounds. Although understanding of the impact of paraben exposure is in the early stages, in vitro and animal studies have reported that parabens demonstrate estrogenic activity with the ability to stimulate growth in human breast cell lines [11–13]. Various studies with lab rodents have reported that exposure to parabens are associated with significantly increased uterine weights in females and significantly decreased testosterone production and altered reproductive tracts in males [7, 10].

Concern has been expressed that parabens alone and/or synergistically in association with other common endocrine disrupting cosmetic ingredients including triclosan, phthalates, and benzyl benzoate, may facilitate estrogenic impact and thus increase the risk of breast cancer and other estrogen sensitive tumors [13]. Some in vitro studies report that exposures to combinations of low levels of multiple varieties of parabens have estrogenic activity significantly exceeding the sum of estrogenic effects of parabens administered individually [13].

Two studies of human breast tumors reported tumor paraben concentrations which were comparable or even slightly below concentrations in which parabens were reported to stimulate growth of human breast cancer cell lines [11, 14]. No measurements of paraben levels in healthy breast tissues, however, were reported in these studies. More epidemiological study is required to determine if the association between parabens and breast cancer is causal or coincidental [13].

Emerging evidence suggests that some types of human paraben exposure might exert hormonal impact on the body. High urinary BP levels were associated with significantly higher rates of sperm DNA breakage [15] and a study of 1,831 US children and adults reported that higher urinary levels of EP was associated with significantly lower levels of thyroxine [16]. Urinary paraben levels were not found, however, to be associated with changes in age of menarche in a study of 440 adolescents [17] and a study of 190 male infertility patients reported that urinary levels of MP, PP, or BP were not associated with significant alteration of conventional sperm quality parameters [15].

Cosmetic paraben exposure may be related to allergic conditions such as contact dermatitis in a small minority of cases. A study of 121,247 European adults reported that 1,752 (1.33%) had positive skin patch tests to a 16% paraben mix [18]. A study of 859 US youngsters aged 6 to 18 years reported that higher urinary levels of BP and PP were associated with significantly higher rates of aeroallergen sensitization [19]. Parabens are also suspected to play a role in the modern obesity epidemic. A study with murine cell cultures reported that parabens promote development and differentiation of multipotent cells into mature adipocyte (fat) cells [20].

As a result of increasing concern about the potential adverse effects of parabens, various companies and jurisdictions have instituted efforts to diminish exposure. For example, some cosmetic companies have recently been producing and promoting paraben-free cosmetics [1] and in 2011, Denmark announced a ban on PP and BP in cosmetic products intended for children under 3 years of age [21].

### 1.3. Population Exposure Levels

At the present time, it is not yet clear what scale of exposure (as indicated by levels of specific urinary parabens) may be directly associated with adverse health outcomes. Furthermore, it is unclear what length of ongoing exposure is required to induce illness. Several studies, however, have reported on urine paraben levels. For example, urinary values for the parabens were measured in several thousand representative subjects in the USA [22]. Population 50% and 95% percentile values for the 4 most common parabens from this National Health and Nutrition Examination Survey (NHANES) by the Centre for Disease Control (CDC) study are summarized in Table 1 for 1,399 males and 1,350 females over the period 2009-2010.

Table 2 summarizes levels of 3 parabens in a group of 105 pregnant women in Puerto Rico [23]. This study reported that higher use of hand/body lotion, sunscreen or mouthwash were all independently associated with significantly higher total combined geometric means of the 3 parabens [23]. Table 2 also summarizes urinary levels of 3 parabens collected in 2,721 samples from 254 men and 408 women from a Massachusetts infertility clinic. This study reported that median MP and PP concentrations were 4.6 and 7.8 times higher in men than women, respectively, and concentrations of both MP and PP were 3.8 times higher in African Americans than Caucasians [24]. Among 129 pregnant women, mean urinary parabens levels were 25–45% lower during pregnancy than before pregnancy [24].

## 2. Materials and Methods

A sample of convenience was done by measuring urinary levels of common parabens in patients at an Alberta primary care clinic specializing in environmental health sciences. Patients presenting for care at this facility generally have diverse concerns and health problems that have not been resolved elsewhere and are generally individuals who have a history of adverse chemical exposures. As part of their clinical care, toxicant assessment and nutritional biochemistry are performed. Because of the expense and lack of availability, a toxicant screen for a plethora of common adverse chemical exposures, as has been reported by the CDC [22], is not usually possible.

When the opportunity presented to have paraben testing done by ALS laboratories, patients presenting for medical care over two days with various problems, were offered the opportunity to provide a urine specimen for paraben testing as part of their clinical assessment. All patients were informed about current knowledge relating to parabens and the potential health concerns related to these compounds. With informed consent, all 39 consecutive patients seen over the 2 day period were keen to participate; none refused. None of the patients were known to have renal problems that might impair their ability to excrete parabens.

Approval was received from the Health Ethics and Research Board at the University of Alberta. A chart review was subsequently performed on all 39 patients who had paraben testing performed and results reported in the summer of 2011. The objective of the study was to compile the results to determine the prevalence of paraben exposure in one representative urban community in Canada and to compare such levels to those found in other jurisdictions.

### 2.1. Sample Collection and Analyses

For urine collection, participants were instructed to collect a mid-stream urine sample directly into a provided 500 mL glass jar container on the same day that blood samples were collected. Each of the glass bottles used for sampling in this study was provided by ALS laboratories and had undergone extensive cleaning and rinsing. The containers were deemed appropriate for urine collection with negligible risk of contamination: laboratory-grade phosphate-free detergent wash; acid rinse; multiple hot and cold deionized water rinses; oven dried; capped and packed in quality-controlled conditions. Urine samples were retrieved by staff of ALS Laboratories on the day of collection. Samples were transferred to 4 mL glass vials and stored in a freezer at −20°C. The urine was then tested for 5 different parabens including MP, EP, PP, BP, and IP.

Urine samples were aliquoted into vials using an Eppendorf epMotion 5075 Automated Pipetting System. Sample aliquots had surrogate, glucuronide solution, and enzyme buffer solution added and were then incubated for 90 minutes. Eighty microliters of 1 M formic acid and 740 microliters of HPLC-grade water were added to each vial, after which the vials were capped and mixed with a Vortex mixer for approximately 10 seconds. The resulting extract solutions were filtered through Captiva filtration plates under vacuum and collected in a 96-deep-well plate.

Filtered samples were transferred to labeled instrument vials with spring inserts and analyzed on a Transcend TLX-2 in-line SPE/HPLC with Thermo TSQ Vantage MS/MS System using a Cyclone 0.5 mm × 50 mm SPE and a Hypersil Gold 50 mm × 2.1 mm 1.9 *μ*m analytical column. Mobile phase for the analysis was a gradient between HPLC-grade water and methanol.

## 3. Results and Discussion

The demographics and the urine paraben levels of the 39 patients tested are listed in Table 3. Means, standard deviations, and ranges of urinary parabens for the subjects and groups of the subjects are listed in Table 4. Tables 3 and 4 reveal that urinary concentration values for the parabens were not normally distributed, with a few very high paraben concentrations. The highest urinary concentrations (in *μ*g/L) reported as 966.46 for MP, 220.6 as EP, and 612.73 for PP. The upper range concentrations of parabens were often many times the average concentrations. Therefore, median urinary paraben values may be most useful in making comparisons between groups of patients in this study and other studies.

**Figure 1:**
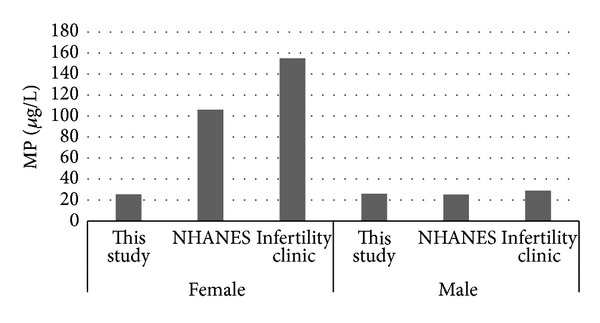
Median urinary MP.

**Figure 2:**
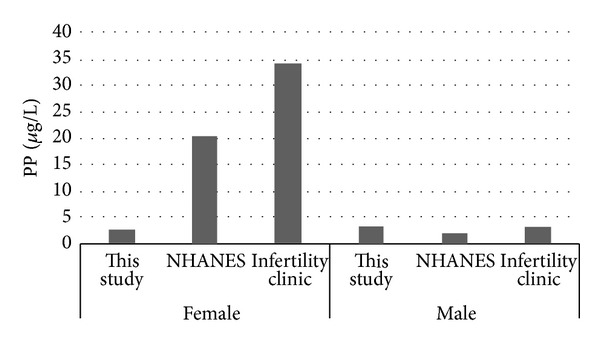
Median urinary PP.

**Figure 3:**
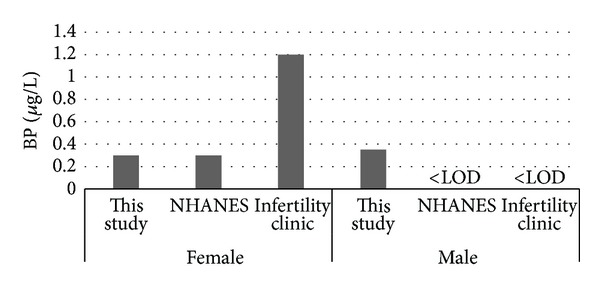
Median urinary BP.

**Figure 4:**
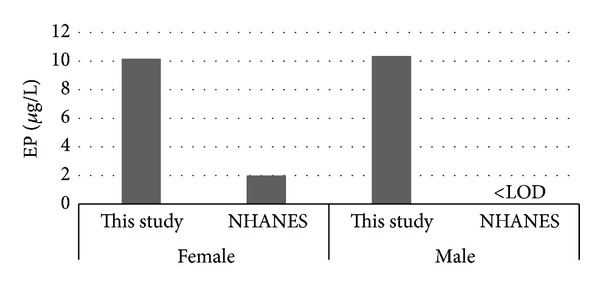
Median urinary EP.

**Figure 5:**
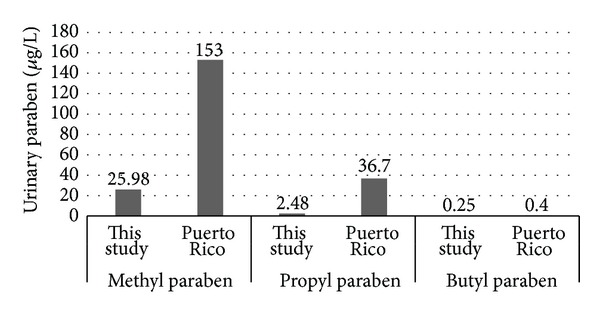
Pregnant women median urinary MP, PP, and BP.

Median levels of MP. PP. BP and EP reported in this study and the results from the NHANES [22] and Infertility Clinic [24] are presented in Figures 1, 2, 3, and 4.  Figure 5 compares median levels of urinary MP, PP, and BP in 105 pregnant women in Puerto Rico [23] and 9 pregnant women in this study.

It is evident from this study that paraben exposure in urban Alberta is a routine event, with various types of parabens found in the urine of all participants to varying degrees. As in other studies, the most abundant paraben found overall in urine is MP followed by PP. median urinary concentrations of all 5 parabens measured were similar in females and males in our study (Table 4, Figures 1–4). This finding differs from that reported in the NHANES and Infertility Clinic studies which reported much higher levels of all of the tested parabens in females as compared to males [22, 24]. The reasons for this difference are uncertain, but as cosmetic consumer products are the largest source of exposure, the difference may be related to the volume of cosmetics or sunscreen used in urban Alberta, where the climate is generally more wintery than in America or Puerto Rico.

Among females, the median levels of urinary MP, PP, and BP were much lower than seen in the NHANES or Infertility Clinic studies [22, 24]. On the other hand, urinary EP levels were much higher in both females and males in this study as compared to the NHANES study [22]. No explanation is offered for this finding. It is unclear if a gestational state has any impact on the excretion of parabens, and whether this state might account for some of the difference in relation to the Infertility Clinic study.

Among pregnant women, much higher levels of urinary MP and PP were seen in the Puerto Rico study as compared to this study [23]. The Puerto Rico study reported significantly higher levels of urinary total parabens (sum of EP, PP, and BP) in pregnant women with higher cosmetic usage. This suggests that higher cosmetic usage may be a factor in the much higher urinary MP and PP levels seen in the Puerto Rico pregnant women versus the pregnant women in this study.

It is alleged by some that at recommended regulatory concentrations, methyl and ethyl parabens have no adverse hormonal impact in humans [25]. The relative hormonal potency of the remaining parabens, however, is still being investigated. With some degree of uncertainty about the risk associated with each paraben, some jurisdictions have endeavored to minimize population exposure by providing regulatory levels to restrict individual paraben concentrations. Various groups including the US Food and Drug Administration (FDA), the European Scientific Committee on Consumer Products (SCCP), and Health Canada have thus far concluded that parabens can be safely used in cosmetic products at low concentrations [5, 26, 27]. While the SCCP provides a legal limit of 0.4% for any individual paraben and 0.8% for total paraben concentrations [26]. North American recommendations are only guidelines and manufacturers are not required to follow them.

Although individual parabens are sometimes used alone, most often various parabens are incorporated in products in the form of a mixture. Emerging evidence confirms that the functional impact and potential risk of the presence of parabens in human tissues may be a factor of the cumulative total of all five parabens used rather than single parabens individually [5]. It is thus recommended that future epidemiological work incorporate measurement of the cumulative level of parabens per product and that population biomonitoring convey aggregate measurements. Because of differences in relative impact of various parabens, however, with some paraben compounds such as BP being considerably more hormonally potent than other individual parabens [28], a reliable biomarker of total functional impact should incorporate both concentration and potency.

As is evident from this study, many pregnant women also harbor parabens with unknown impact on the developing child. Previous work has demonstrated an apparent accumulation of parabens within the amniotic fluid [29] thus raising concern about the margin of safety for paraben exposure during pregnancy. Human studies exploring cord blood levels and associated long term impact are required. This study is being followed up by Alberta Health with an assessment of approximately 250 serum samples from pregnant women with associated cord blood samples. Furthermore, Health Canada has begun to submit in the range of 1700 urine samples from Canadians for paraben assessment.

It has generally been believed that parabens do not accumulate in the body [5]. With a decline in exposure, serum concentrations of parabens, even after intravenous administration, quickly decline and remain low. However, recent studies have found parabens in breast milk, seminal fluid, and the breast tissue from patients with breast cancer [5, 11, 14, 30–32]. There is uncertainty whether, like some other chemical compounds, parabens may bioaccumulate and persist in tissues without evidence of their presence in serum after the acute exposure period has passed.

## 4. Conclusion

The desire to have germ-free consumer products with long shelf-lives has necessitated the use of preservatives. The optimal preservative for such use should be effective at low concentrations with potency against a wide range of micro-organisms. It should be toxic to neither humans nor the environment, and should not hinder the action of the product being preserved. Parabens initially appeared to fulfill these criteria and were thus incorporated without reservation into a variety of personal care, cosmetic, pharmaceutical, and food products many years ago.

Recent evidence, however, has demonstrated contamination of the environment with detection of parabens in rivers and drinking water, agricultural soil, wastewater, as well as indoor dust and air [33–37]. Furthermore, these compounds have been found to have hormone disrupting action and have been detected in human tissues such as breast tissue as well as some bodily fluids.

This study confirms that the whole range of synthetic parabens commonly used as preservatives are found routinely in both men and women, including all pregnant women tested, in an urban area of Alberta, Canada. As the study of exposure science is very new on the continuum of scientific knowledge, there are many uncertainties about the long term impact of such paraben exposure on the human organism, about possible synergism with other toxicants and about apparent carcinogenicity, potential teratogenicity, and details regarding toxicokinetics and possible tissue bioaccumulation. It is recommended that increased awareness of parabens be facilitated and that a precautionary approach to exposure be taken until firm evidence of safety is available.

## Figures and Tables

**Table 1:** Urinary concentrations of parabens reported from 2009-2010 NHANES study [22]. All units are *μ*g/L.

Paraben	1,350 females—all ages		1,399 males—all ages	
	50% percentile	95% percentile	50% percentile	95% percentile
Methyl paraben	106	1,230	25.3	727
Ethyl paraben	2.00	138	<LOD	36.4
Propyl paraben	20.2	361	2.00	134
Butyl paraben	0.300	31.8	<LOD	2.70

<LOD means below limit of detection.

**Table 2:** Urinary concentrations of parabens reported from Puerto Rico Protect study [23] and Infertility clinic patients [24]. All units are *μ*g/L.

Paraben	Puerto Rico—105 pregnant women aged 18–40 years [23]		Infertility clinic—408 women aged 20.9–46.7 years [24]		Infertility clinic—245 men aged 23.9–56.8 years [24]	
	50% percentile	95% percentile	50% percentile	75% percentile	50% percentile	75% percentile
Methyl paraben	153	1,590	155	422	29	96.7
Propyl paraben	36.7	493	34.3	118	3.1	16.8
Butyl paraben	0.4	36.4	1.20	7.65	<LOD	0.50

<LOD means below limit of detection.

**Table 3:** Demographics and urinary levels of parabens in the 39 patients in this study. All paraben units are ng/mL.

Sample ID no.	Gender	Age	Pregnant	Methyl paraben	Ethyl paraben	Propyl paraben	Butyl paraben	Isobutyl paraben
1	F	50	no	6.16	8.87	5.13	1.83	0.22
2	M	17	no	25.95	10.57	3.86	0.14	0.44
3	F	21	no	8.91	9.56	1.96	0.14	0.14
4	F	44	no	109.65	46.56	25.12	2.80	2.58
5	F	49	no	3.25	8.79	1.27	0.49	0.54
6	F	20	yes	15.89	8.95	2.47	0.14	0.14
7	F	20	no	212.15	18.13	42.56	0.55	0.28
8	M	19	no	196.37	29.23	5.84	0.44	0.34
9	M	65	no	15.27	9.69	3.09	0.43	0.22
10	F	37	no	11.79	11.89	1.48	0.14	0.14
11	F	21	yes	44.75	7.82	2.48	0.14	0.14
12	F	26	yes	11.24	9.83	1.89	0.27	0.25
13	F	19	yes	409	29	35.7	22.9	0.54
14	F	46	no	52.75	29.26	4.26	0.87	0.55
15	F	46	no	14.01	8.25	1.26	0.14	0.14
16	F	27	yes	25.98	10.04	2.80	0.25	0.27
17	F	52	no	44.38	13.34	2.71	0.21	0.14
18	M	40	no	49.68	9.42	2.69	0.16	0.14
19	F	67	no	270.39	62.44	130.17	10.92	9.70
20	M	26	no	16.48	9.21	2.76	0.51	0.21
21	F	23	yes	10.81	10.73	2.06	0.16	0.14
22	F	25	yes	97.24	10.30	2.91	0.24	0.14
23	F	43	no	966.46	220.63	297.94	5.99	6.42
24	M	40	no	102.39	12.38	6.23	1.00	0.38
25	F	37	no	11.52	5.29	2.59	0.25	0.56
26	F	41	no	3.88	8.15	2.58	0.32	0.14
27	F	55	No	107.00	31.58	11.55	3.24	2.93
28	F	44	No	279.38	43.80	131.30	3.84	3.97
29	M	55	No	13.67	15.89	2.02	0.35	0.16
30	M	31	No	16.24	10.37	2.57	0.40	0.31
31	F	45	No	24.92	9.46	15.96	0.14	0.17
32	M	12	No	65.15	8.67	37.01	0.23	0.14
33	F	23	Yes	29.79	7.54	3.13	0.49	0.21
34	F	59	No	32.84	20.45	3.18	0.71	0.31
35	F	62	No	10.17	7.74	2.79	0.29	0.23
36	M	45	No	534.62	9.84	612.73	0.14	0.74
37	F	56	No	18.64	10.99	1.16	0.17	0.25
38	M	60	No	15.06	16.25	1.70	0.14	0.14
39	F	33	Yes	4.90	8.84	1.33	0.51	0.14

**Table 4:** Means, standard deviations, and ranges of urinary parabens in the 39 subjects. All paraben units are ng/mL.

Group	No. of patients	Statistic	Methyl paraben	Ethyl paraben	Propyl paraben	Butyl paraben	Isobutyl paraben
All patients	39	Mean	99.7	21.0	36.4	1.59	0.89
		Std dev.	184.7	35.0	109.1	4.04	1.91
		50% percentile	25.95	10.30	2.80	0.32	0.23
		Low	3.25	5.291	1.16	0.14	0.14
		High	966.46	220.6	612.73	22.9	9.7

All female patients	28	Mean	101.3	24.4	26.45	2.08	1.12
		Std dev.	197.4	40.9	63.1	4.7	2.22
		50% percentile	25.45	10.17	2.80	0.30	0.24
		Low	3.25	5.291	1.16	0.14	0.14
		High	966.46	220.63	297.94	22.9	9.7

Pregnant women	9	Mean	72.1	11.45	6.09	2.79	0.22
		Std dev.	129.3	6.67	11.11	7.54	0.130
		50% percentile	25.98	9.83	2.48	0.25	0.14
		Low	4.9	7.54	1.33	0.14	0.14
		High	409	29	35.7	22.9	0.54

Non pregnant women	19	Mean	115.2	30.28*	36.0	1.74	1.55
		Std dev.	224.6	48.7	75.0	2.75	2.61
		50% percentile	24.92	11.89	3.18	0.49	0.28
		Low	3.25	5.291	1.16	0.14	0.14
		High	966.46	220.63	297.9	10.92	9.7

Men	11	Mean	93.53	12.87	61.9	0.36	0.29
		Std dev.	155.9	6.01	182.0	0.254	0.181
		50% percentile	25.95	10.37	3.09	0.35	0.22
		Low	13.67	8.67	1.7	0.14	0.14
		High	534.62	29.23	612.73	1.0	0.74

*Denotes that mean paraben value significantly different from male values (baseline) via a *t*-test.
